# Complete genome sequences of soil-associated *Paenibacillus* spp. SEL1, SEL2, and SEL3

**DOI:** 10.1128/mra.00398-26

**Published:** 2026-05-29

**Authors:** Thao D. Tran, SangIn Lee, Robert Hnasko, Jeffery A. McGarvey

**Affiliations:** 1 Foodborne Toxin Detection and Prevention Research Unit, Agricultural Research Service, U.S. Department of Agriculture 230648, Albany, California, USA; 2 Produce Safety and Microbiology Research Unit, Agricultural Research Service, U.S. Department of Agriculture 551257 , Albany, California, USA; Nanchang University, Nanchang, Jiangxi, China

**Keywords:** *Paenibacillus*, soil microbiology, Salinas Valley, lettuce farm

## Abstract

Three *Paenibacillus* spp., designated SEL1, SEL2, and SEL3, were isolated from soil samples collected at lettuce farms in the Salinas Valley of California in fall 2020. Genomic sequencing of these isolates was performed using the PacBio Revio platform. All three genomes possess circular whole-genome sequences and lack detectable plasmids.

## ANNOUNCEMENT

*Paenibacillus* species are ubiquitous in soil and have been documented to enhance plant growth and for biocontrol of pathogens (1–3). Three *Paenibacillus* spp. strains, designated SEL1, SEL2, and SEL3, were isolated from agricultural soils near Salinas, CA (approximate coordinates 36.4357° N, 121.3653° W) as described previously (4). These strains were found to suppress the persistence of *Salmonella enterica*, *Escherichia coli* O157:H7, and *Listeria monocytogenes*, utilizing the method developed by Devarajan et al. (5).

Soil samples collected from between lettuce rows were diluted and blended in phosphate-buffered saline before being cultured on Reasoner’s 2A agar (BD Difco, MA) for SEL1 and SEL2 and tryptic soy agar (TSA) (BD Difco, Massachusetts) for SEL3. All plating media were supplemented with 40 mg/L cycloheximide and incubated at 25°C for 3 days. Individual colonies were isolated and consecutively passaged three times on TSA, with a final streaking on TSA. For genomic DNA extraction, a single colony was cultivated overnight in tryptic soy broth for 24 h at 37°C with continuous shaking at 200 rpm and then harvested. DNA was extracted using a sucrose-Tris method incorporating phenol-chloroform cleanup, as previously described (6), resulting in a total yield of ~20 µg. Of this, 5 µg of genomic DNA underwent shearing with a Covaris G-tube (Covaris, Massachusetts) in an Eppendorf MiniSpin plus microcentrifuge (Eppendorf, Connecticut) at 3,200 × *g*, targeting fragment lengths of 10–12 kb. No subsequent size selection was applied to these fragments. Whole-genome SMRTbell libraries for SEL1, SEL2, and SEL3 were prepared following the SMRTbell prep kit 3.0 procedure and checklist, barcoded using SMRTbell barcoded adapter plate 3.0 (PacBio, Menlo Park, CA), and sequenced in a single-molecule real-time (SMRT) cell. This sequencing utilized PacBio Revio SPRQ chemistry (v13.3.0.249246), with an on-plate loading concentration of 300 pM and standard HiFi library loading. The PacBio Revio run yielded a total of 12,036,608 raw reads, which included data from three other genomes not reported here. The raw data were then demultiplexed and converted to HiFi reads files using microbial genome analysis (SMRT Link v25.2.0.266456, PacBio). The HiFi reads corresponding to each bacterium were employed to assemble and circularize chromosomal contigs using Hifiasm 0.25.0-r726 (10% of the total reads) (SCINet project of the USDA Agricultural Research Service), confirmed by microbial genome analysis (total number of reads) (SMRT Link v25.2.0.266456, PacBio). Manual inspection for circularity using the detection of overlapping ends and trimming was performed using Geneious Prime (v2026.0.2), confirming the presence of a single circular chromosome with no additional circular contigs for each bacterium (Table 1); no plasmids were detected in this specific data set, though small plasmids outside the selected size range cannot be entirely ruled out. Genome rotation was not performed. An internal sequencing control included in the PacBio Revio SPRQ Polymerase Kit (PacBio) was used, demonstrating a base quality Q30 of 95.27%. The assembled genomes were submitted to the NCBI Prokaryotic Genome Annotation Pipeline (v6.10) for annotation (7–9), with default parameters applied for all software.

**TABLE 1:** Genomic features of *Paenibacillus* species SEL1, SEL2, and SEL3^*a*^

Characteristics	SEL1	SEL2	SEL3
Genome sequencing output per run			
Total bases generated (bp)	16,115,736,581	16,495,110,596	17,230,475,792
Demultiplexed total HiFi reads	1,659,624	1,695,915	2,056,487
HiFi read quality (median)	Q40	Q40	Q40
HiFi read length N50 (bp)	9,676	9,676	9,676
Genome assembly			
Total HiFi reads used	1,659,624	1,695,915	2,056,487
Mapped HiFi reads	1,659,107	1,695,343	2,055,627
Mapped HiFi read length N50 (bp)	10,725	10,757	9,207
Contig(s)	1	1	1
Genome characteristics			
Genome size (bp)	5,761,366	5,761,366	5,812,254
Coverage (×)	2,822	2,888	2,907
GC content (%)	45.7	45.7	45.4
Circular	Yes	Yes	Yes
Coding sequences	5,084	5,108	5,750
Coding genes	4,982	5,010	5,638
Closest identified reference (% similarity)	CP015423 (88.5)	CP015423 (88.5)	CP092831 (79.7)
Data availability			
GenBank accession number	JBTSVT000000000	JBTSVU000000000	JBTSVV000000000
SRA accession number	SRR36399349	SRR36399348	SRR36399347
BioProject number	PRJNA1377667	PRJNA1377667	PRJNA1377667
BioSample number	SAMN53797586	SAMN53797587	SAMN53797588

^*a*^ CCS, circular consensus sequencing; SRA, Sequence Read Archive.

**Fig 1:**
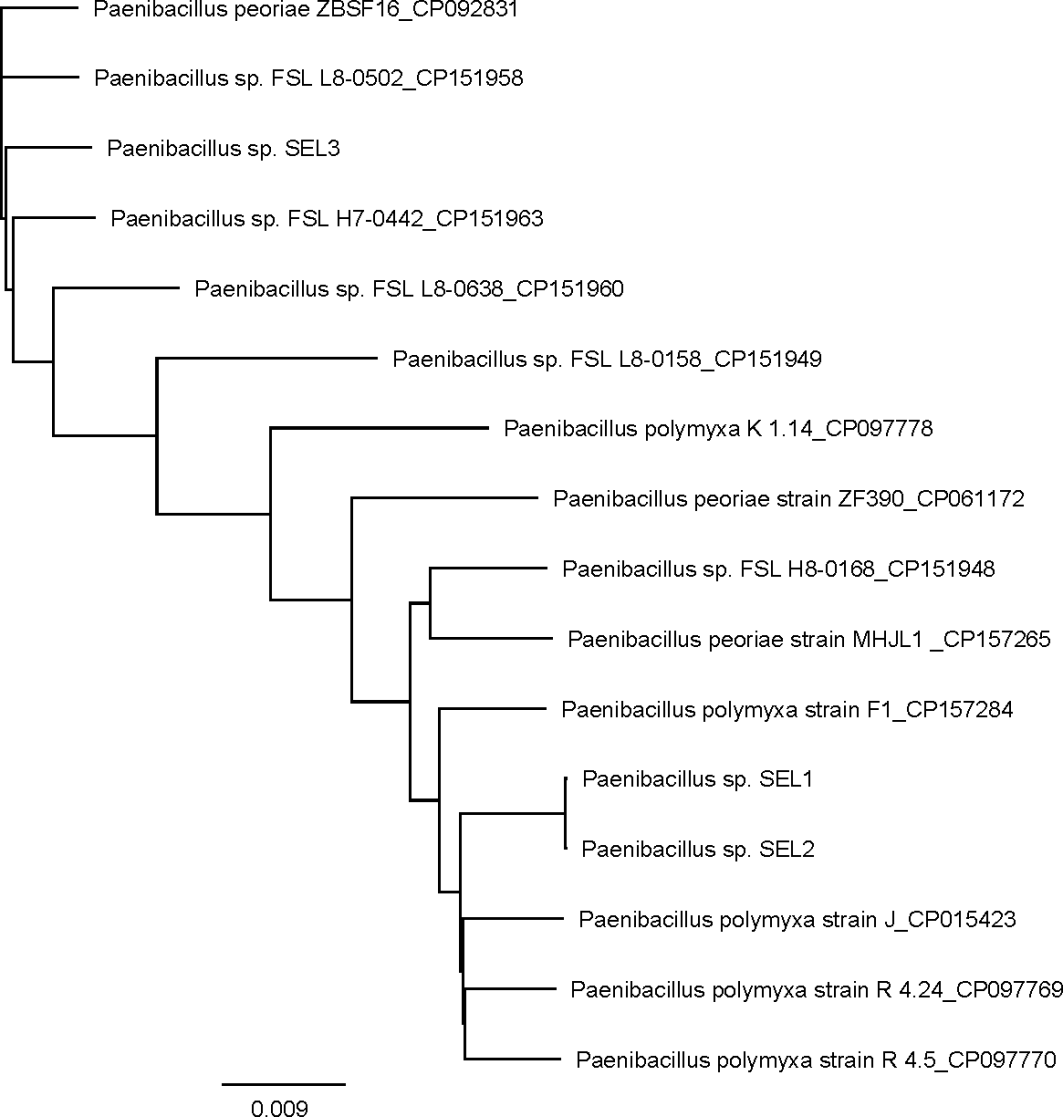
Comparative analysis was conducted to assess similarities among *Paenibacillus* spp. strains SEL1, SEL2, and SEL3, and other *Paenibacillus* spp. strains available on GenBank. This assessment was performed utilizing the mauve whole-genome aligner (Geneious Prime v2026.0.2). Similarity percentages were calculated by counting the nucleotides aligned between two genomes in the initial local multiple alignments, then dividing that shared count by the average genome size to get a value from 0 to 1. Overlaps among matching regions are removed so each aligned residue is counted only once. Subsequently, a phylogenetic tree was constructed using the distance-based neighbor-joining method, incorporating the Tamura-Nei model. Bootstrapping was applied with 100 replicates and a support threshold of 0 (Geneious Prime v2026.0.2). Three 200 kb fragments sampled from the beginning, middle, and end of the genome sequence were queried against the NCBI nucleotide database using BLAST, and the top five hits for each fragment were evaluated; reference genomes were selected only when all three fragments showed 100% identity and >95% query coverage. The complete bacterial genomes were imported into Geneious directly from NCBI. Unless otherwise specified, default parameters were applied throughout the analysis.

A detailed overview of the characteristics for the SEL1, SEL2, and SEL3 genome assemblies is presented in Table 1. These bacteria were classified within *Paenibacillus* by 16S rRNA gene sequence analysis (data not shown). Taxonomic resolution was limited to the genus level. To determine their genetic relationships, a similarity analysis was conducted using Geneious (v2026.0.2), comparing the genome sequences of *Paenibacillus* spp. SEL1, SEL2, and SEL3 with other *Paenibacillus* spp. sequences publicly available in GenBank. The results of this analysis are illustrated in Fig. 1. The genome sequences for *Paenibacillus* spp. SEL1, SEL2, and SEL3 have been deposited in DDBJ/ENA/GenBank, and their HiFi raw reads are accessible via the Sequence Read Archive, with all relevant accession details listed in Table 1.

## Data Availability

This Whole Genome Shotgun project has been deposited in DDBJ/ENA/GenBank under the accession numbers shown in Table 1. The version described in this paper is the first version.
